# Influence of the Quality Perceived of Service of a Higher Education Center on the Loyalty of Students

**DOI:** 10.3389/fpsyg.2021.671407

**Published:** 2021-06-07

**Authors:** María de la Cruz Del Río-Rama, José Álvarez-García, Nam Kwon Mun, Amador Durán-Sánchez

**Affiliations:** ^1^Business Management and Marketing Department, Faculty of Business Sciences and Tourism, University of Vigo, Ourense, Spain; ^2^Financial Economy and Accounting Department, Faculty of Business, Finance and Tourism, University of Extremadura, Cáceres, Spain; ^3^Department of Latin American Studies, Graduate School of International and Area Studies (GSIAS), Hankuk University of Foreign Studies, Seoul, South Korea

**Keywords:** perceived quality, perceived value, satisfaction, loyalty, higher education

## Abstract

The aim of this research is to validate the explanatory model of how the quality of service perceived by students of a higher education center influences their loyalty (retaining and attracting new students) through mediating variables: perceived value, expectations, and satisfaction. The methodology used to validate the measurement scales is exploratory, and confirmatory factor analysis and the structural equation modeling (SEM) technique are applied to analyze the causal relationships proposed in the model. The results show that the key variables to improve student's loyalty to the center are the quality of the service provided and the satisfaction perceived by the students. Both variables are postulated as a major source of competitive advantages. It is also observed that service quality is one of the three key variables to achieve student's satisfaction together with expectations and perceived value. This research and its results allow us to understand the relationship between quality and satisfaction with loyalty and to identify the background variables of satisfaction (perceived service quality, perceived value, and expectations), as well as to obtain evidence of the importance that expectations have within the model for the formation of both perceived quality and satisfaction.

## Introduction

In recent years, the university has undergone major changes, including, in general terms, globalization (Maringe and Gibbs, 2009; Altbach et al., 2010), the internationalization level that allows for free movement of students (Altbach, 2004), increased competition from the private sector, reduced funding (Hemsley-Brown et al., 2016; Verčič et al., 2016), and demographic causes, such as low birth rates experienced by many countries (Maringe and Gibbs, 2009). These changes lead to the university being immersed in highly competitive, global, and highly changing markets, competing for students, resources (human and financial), and reputation/image. In this context, the student becomes the focus of attention of universities, and strengthening the relationship with their students is the key for future success (Fernández et al., 2007), thus retaining current students and attracting new students.

In this regard, there are many studies that show that proper management of the intangible assets owned by organizations (university) leads to achieving a better competitive position (Lev and Zarowin, 1999; Hand and Lev, 2003) and achieving both their social and economic objectives in the medium and long term (Farrugia and Lane, 2013; Hemsley-Brown et al., 2016; Plewa et al., 2016; Christensen and Gornitzka, 2017). Thus, these intangible assets become key elements for survival. In this context, universities must be concerned about aspects that are closely linked to their survival, such as the background dimensions of loyalty, service quality, perceived value, expectations, and satisfaction, among others.

Service quality is a key intangible asset for achieving student's loyalty that must be managed to create a competitive advantage and differentiate itself from the competition in current markets (Paramewaran and Glowacka, 1995), in order to attract and retain/loyalty from the best students (Helgesen and Nesset, 2007; Stevens et al., 2008; Polat, 2011). It is clear that what currently determines the competitive position of a company or organization, as well as its long-term survival “is customers' opinion regarding the product or service they receive” (Aquino and Vogel, 2009, p. 1), that is, the quality perceived.

The literature on perceived service quality is very extensive; empirical studies focused on how to measure service quality in organizations, as well as studies that seek to understand the relationships between service quality, satisfaction, and loyalty (Parasuraman et al., 1985, 1988; Cronin and Taylor, 1992; Fornell, 1992; Iacobucci et al., 1995; Oliver, 1999; Cronin et al., 2000; Dabholkar et al., 2000; Mahamad and Ramayah, 2010). However, few studies show the consequences of quality education (Chua, 2004; Blass and Weight, 2005; Cornuel, 2005; De Oliveira and Ferreira, 2009) and study the relationship between service quality and loyalty in the higher education sector (Rowley, 1996; Peng and Samah, 2006; Tsuji et al., 2007; Yunus et al., 2010). This research specifically considers the direct relationship between both constructs, as well as the indirect relationship through variables, such as satisfaction, expectations, and perceived value. This research contributes to the existing knowledge by providing empirical evidence of how the antecedents considered in the proposed model have an influence, and in particular, quality on student's loyalty in the higher education sector, enabling to strengthen the relationship between the university and the student.

Following this line of work, the objective is to contrast an explanatory model of how the quality of the service perceived by the students of a university center influences their loyalty to the center through variables, such as perceived value, expectations, and satisfaction. The methodology used to validate the proposed model is the structural equation modeling (SEM) technique. The target population is students of a university faculty in Spain, and 224 valid surveys were obtained through a structured questionnaire.

It is important to analyze the structure of relationships between loyalty and service quality since “it will allow university managers to know what dimension/s of quality to focus their efforts on, such as increasing the perceived value of students, how to manage expectations to improve their students' satisfaction and consequently to increase their loyalty to the center. This knowledge will allow them to implement appropriate programs that promote, establish, develop and maintain successful long-term relationships with current and former students” (Annamdevula and Bellamkonda, 2016, p. 446).

This paper is structured into five sections. In the introduction, the subject under study is contextualized and the research is presented together with the objective. The theoretical framework that supports the research is explained in the following section, and the working hypotheses are presented. In section Methodology, the methodology is described; sample, questionnaire, and data analysis and the empirical results are presented in section Results. Finally, the results are discussed, the main conclusions are drawn, and the limitations of the research are explained.

## Literature Review and Hypotheses

### Perceived Service Quality

The concept of perceived service quality arises when considering quality from an approach focused on technical aspects (objective quality focused on the service provider perspective); it evolves toward a more subjective approach based on customer perceptions (external or provision dimension). It is a more appropriate approach in the context of services. Its representatives are the North American School led by Parasuraman, Zeithaml, and Berry, who propose that perceived service quality should be defined from the customer perception perspective, focusing on the delivery phase of quality service (Parasuraman et al., 1985).

On the other hand, this concept is very complex and vague due to the intrinsic characteristics of services (intangibility, separability, expiration, etc.): (1) it is difficult to evaluate service quality, as it is necessary to evaluate intangible aspects that are difficult to identify and quantify (Parasuraman et al., 1985) and (2) the aspects are liable to different evaluations by their clients (Zeithaml, 1988; Rosenbloom, 1991). According to Parasuraman et al. (1985, p. 36), “the difference between the evaluation of the quality of a service and that of a good by a consumer is not in the process, but in the nature of the characteristics on which the evaluation is made.” In the higher education context, it becomes even more complex, according to Annamdevula and Bellamkonda (2016, p. 447), due to some unique characteristics, such as (1) the cognitive participation of students in the service process, (2) students' needs that are satisfied by different parties, (3) continuous services, and (4) long-term services.

All this leads to numerous definitions being proposed by experts in the field (see Grönroos, 1982, p. 33; Zeithaml, 1988; Carman, 1990, p. 33; Koelemeijer et al., 1993). As in the case of the service sector, in the educational context, Harvey and Green (1993) state that it is a multitasked concept and lacks a correct definition. Therefore, there is also a lack of consensus in this sector on how to define and measure service quality (Clewes, 2003; Sultan and Wong, 2012). However, there is consensus that students are the priority clients of educational activities (Gremler and McCollough, 2002; Marzo-Navarro et al., 2005). This research considers the proposal by O'Neil and Palmer (2004, p. 40) for the university education sector, “the difference between what a student expects to receive and his/her perceptions of the actual delivery.” To propose it, these authors reviewed the current literature and support the ideas proposed by Parasuraman et al. (1988), who define it as a global judgment of the consumer regarding the superiority of a service, which results from the comparison made by clients between the expectations regarding the service they are going to receive and the perceptions of the performance of the organizations providing the service (Parasuraman et al., 1985; Grönroos, 1994). Therefore, a service can be said to be of quality when it meets or exceeds the client's expectations (Grönroos, 1990; Zeithaml, 1990).

As it has been revealed, to assess the perceived quality of a service by a client, it is necessary to identify which dimensions are considered for its assessment. Currently, there is a consensus about the multidimensional nature of the concept, but not in the number or content of the dimensions that make up perceived quality (Parasuraman et al., 1991). Specifically, in the university education sector, LeBlanc and Nguyen (1997) consider seven dimensions (personal contact with teachers, reputation, physical evidence, personal contact with administrative staff, curriculum, responsiveness, ease of access). Li and Kaye (1998) use the five dimensions proposed by Parasuraman et al. (1988) for the service sector (tangibility, reliability, security, empathy, and responsiveness). Owlia and Aspinwall (1996) propose four dimensions (attitude, content, academic resources, and competence). Kwan and Ng (1999) consider seven dimensions (course content, facilities, assessment, advisory service, communication with the university, teachers' concern about students, and social activities). Oldfield and Baron propose to use essential elements (requirements), desirable elements (aspects), and functional elements. In this research, the scale of De la Fuente Mella et al. (2010), which was created based on an extensive literature review (Cuthbert, 1996; LeBlanc and Nguyen, 1997; Owlia and Aspinwall, 1998; Kwan and Ng, 1999; Alves, 2000; Oldfield and Baron, 2000; Cardone et al., 2003; Alves and Raposo, 2004; Marzo-Navarro et al., 2005), is considered and adapted. It is made up of five dimensions (facilities, service staff, teachers' attitudes and behavior, competence of teachers, and career opportunity).

In this context, the following hypothesis is proposed:

H1: The perceived service quality of the Faculty is a multidimensional construct made up of the facilities, service staff, teachers' attitudes and behavior, competence of teachers, and career opportunity dimensions.

### Consequences of Perceived Service Quality

#### Relationship of Perceived Quality With Loyalty

Oliver (1997, p. 392) define loyalty as “*… a deeply held commitment to rebuy or repatrionize a preferred product or service consistently in the future, despite situational influences and marketing efforts having the potential to cause switching behavior*.” This same author affirms that there are four loyalty phases and suggests that customers can become loyal in any of these phases: (1) cognitive loyalty, attitude toward the brand based on the information provided; (2) affective loyalty, attitude toward the brand due to its successful repeated use; (3) conative loyalty, related to the customer's behavioral intention toward a repeat purchase; and (4) loyalty, additional desire to overcome obstacles that could prevent a repeat purchase. Along these same lines, Dick and Basu (1994) and Lam et al. (2004) consider that loyalty is made up of two interrelated components, namely, the relative attitude (linked to components 1, 2, and 3) and the repeat purchase pattern (retention of repeated client sponsorship). Hennig-Thurau et al. (2001) and Navarro et al. (2005) take this vision into account to define student's loyalty in the higher education context. Therefore, they consider that student's loyalty has an attitudinal component and a behavioral component. This loyalty is configured as the establishment of long-term relationships between the university and its current and former students.

In the university education context, there are studies that corroborate the positive relationship between perceived quality (high quality) and the loyalty of its students (Boulding et al., 1993; Zeithaml et al., 1996; Helgesen and Nesset, 2007). According to Hennig-Thurau et al. (2001), the latter is considered both before and after the completion of student's studies. Other studies also corroborate that there is not only a direct but also an indirect relationship through other variables, such as satisfaction (Bloemer et al., 1999; Caruana, 2002; Huili and Jing, 2012). Therefore, the following hypothesis can be proposed:

H2: The perceived quality of the Faculty influences loyalty toward it directly and positively.

#### Relationship of Perceived Quality With Satisfaction Antecedents: Perceived Value and Expectations

There are many studies carried out in the service sector in which the concepts of service quality, satisfaction, and loyalty are related (Nguyen, 2009), with a consensus in their interrelations, in the sense that if service quality is improved, as a determining/background element of satisfaction (Ahmed et al., 2010; Clemes et al., 2013), satisfaction also improves (Bloemer et al., 1999; Gronholdt et al., 2000; Caruana, 2002; Mahamad and Ramayah, 2010; Huili and Jing, 2012; Olsen et al., 2012), and in compensation, loyalty increases (Annamdevula and Bellamkonda, 2016). However, at present, although the mediating role of satisfaction in service quality and loyalty relationship was corroborated in numerous studies (Huili and Jing, 2012; Jiewanto et al., 2012; Clemes et al., 2013), there is no consensus on what the satisfaction antecedents are and their relationship with quality. Three types of satisfaction antecedents are considered in this research: perceived quality, perceived value, and students' expectations.

##### Perceived Value

Perceived value is a key element in the management of services (Cronin et al., 2000), by enabling to create a competitive advantage (Woodruff, 1997), based on its ability to analyze and predict consumer behavior (Huber et al., 2007). There are many approaches followed to conceptualize this concept, as it is very ambiguous and subjective (a different perception for each client) (Flint et al., 2002; Wang et al., 2004). One of the first definitions was the one proposed by Zeithaml (1988, p. 13), “the global assessment that the consumer makes of the usefulness of a product based on the perceptions of what is delivered and received.” Therefore, it implies reaching a balance between the benefits that the client obtains and the sacrifices made to acquire the service (Zeithaml, 1988; McDougall and Levesque, 2000; Hermawan, 2001; Ledden et al., 2007). In the higher education field, Hermawan (2001), LeBlanc and Nguyen (1999), and Ledden et al. (2007) follow Zeithaml's perspective and consider that it is the general assessment made of the usefulness of the service, based on the perception of what is received and what is given.

In the higher education context, there is little research that focuses on the analysis of value creation toward students (LeBlanc and Nguyen, 1999; Ledden et al., 2007; Suki et al., 2008; Brown and Mazzarol, 2009; Floyd et al., 2009; Yeop et al., 2009; Alves, 2010; Lai et al., 2012). However, in the literature on services, it is analyzed confirming a direct impact of perceived quality on perceived value (Brady and Robertson, 1999; Teas and Agarwal, 2000; Tam, 2004), and this, in turn, influences the client's satisfaction positively (Heskett et al., 1997; Tam, 2004). In this regard, when students perceive that the quality of the service exceeds the costs of obtaining the service, the greater the perception of the value of the service, and in turn, the greater their satisfaction (Tam, 2004).

This leads to the following hypotheses:

H3: The perceived quality of the Faculty influences the value perceived by the student directly and positively.

H4: The perceived value of the Faculty influences student's satisfaction directly and positively.

##### Expectations

They are defined by Parasuraman et al. (1988) as clients' desires or needs. In this regard, it is important for service providers to identify them in order to meet their clients' expectations. In the educational context, students' expectations are the result of previous experiences with similar services, the information received from the education center itself, and friends and family's opinions.

Research carried out in the service sector (Anderson, 1994; Coye, 2004; Hsieh et al., 2011; Gures et al., 2014) confirms that there is a direct relationship with customer satisfaction due to its role as a determinant in the satisfaction assessment process (Parasuraman et al., 1985, 1988; Grönroos, 1994; O'Connor et al., 2000; Pham and Simpson, 2006). Although there are fewer studies in the education sector, this relationship is also corroborated (Alves and Raposo, 2007; Shahsavar and Sudzina, 2017; Marimon et al., 2020). In addition, the relationship between expectations and perceived value and perceived quality is corroborated (Alves and Raposo, 2007; Shahsavar and Sudzina, 2017).

It is expected to have a direct relationship with student's satisfaction.

H5: The student's expectations influence perceived quality directly and positively.

H6: The student's expectations influence perceived value directly and positively.

H7: The student's expectations influence student's satisfaction directly and positively.

#### Relationship of Satisfaction With Loyalty

One of the first definitions of the concept of satisfaction was provided by Oliver (1981, p. 28) for the service sector “as the consumer's value judgment regarding pleasure derived from the utilization of level fulfillment.” In the educational field, a context that is of interest to us, although there is not much literature on this issue (Annamdevula and Bellamkonda, 2016), there is in fact a consensus that considers this concept as complex, which depends on the context of analysis (Giese and Cote, 2000; Navarro et al., 2005).

One of the first definitions is the one proposed by Elliot and Healy (2001), “it is a short-term attitude that results from the assessment of their experience with the educational service received.” Subsequently, it is explained by Elliott and Shin (2002, p. 197) “. the favorability of a student's subjective evaluation of the various outcomes and experiences associated with education” and they further clarify that satisfaction is continuously formed from the student's repeated experiences in the center.

There are many studies that study student's satisfaction with the quality services of educational institutions (Douglas et al., 2006; Sigala et al., 2006; Alves and Raposo, 2007), as well as those that support the relationship between satisfaction and loyalty (Gronholdt et al., 2000; Chaudhuri and Holbrook, 2001). In these studies, satisfaction acts as an antecedent to loyalty (Bitner, 1990), so greater satisfaction causes an increase in loyalty (Fornell, 1992), which results in attracting new students caused by word-of-mouth communication (Clemes et al., 2008, 2013) and in retaining current students (Mavondo et al., 2000; Wiers-Jenssen et al., 2002; Schertzer and Schertzer, 2004; Marzo-Navarro et al., 2005; Helgesen and Nesset, 2007).

H8: The satisfaction perceived by the students of the Faculty influences their loyalty to the Faculty directly and positively.

**Figure 1:**
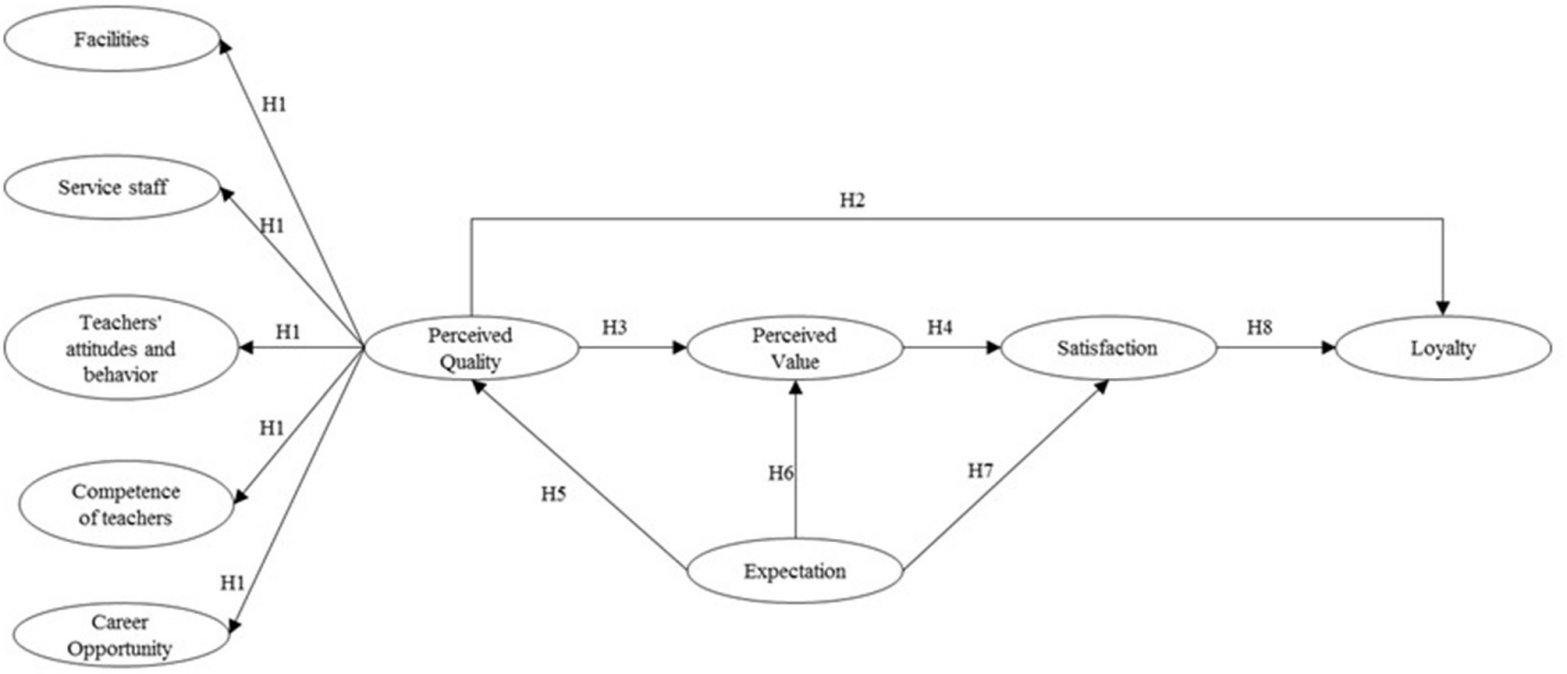
Theoretical model and hypothesis.

To summarize, in Figure 1, the “path diagram” is shown.

## Methodology

### Universe Study, Questionnaire, and Measurement

The target population for the study is students enrolled at a Higher Education Centre in Spain, with a total population of 1,486 undergraduate students studying in the area of knowledge of Tourism, Finance, Accounting, and Business Administration. The means used to collect the data was through an online structured questionnaire (Google Forms), which was active on the platform for 1 month, obtaining a sample of 224 valid questionnaires. The response rate was 15.07%, and the sampling error was 6% for a confidence level of 95% (*Z* = 1.96, *p* = *q* = 0.50). The common method bias (CMB) was verified through the Harman single-factor test (Podsakoff and Organ, 1986); the non-existence of the common method problem in this research was confirmed.

To form the structured questionnaire, the literature was reviewed to identify the measurement scales of the constructs included in the model used by other studies. Specifically, the scale to measure service quality with 40 items was adapted from De la Fuente Mella et al. (2010), while the scale of Schlesinger et al. (2014) was used to measure perceived value, adapted from Dodds et al. (1991). The expectation scale (three items) was obtained from Schlesinger et al. (2014), adapted from Morgan and Hunt (1994), and satisfaction was measured by using the three-item scale by Schlesinger et al. (2014), adapted from Fornell (1992). Finally, the loyalty scale (four items) was from Cervera et al. (2012), adapted from the scale of Martensen et al. (1999) and Hennig-Thurau et al. (2001) (see Appendix). A Likert scale ranging from 1 “totally disagree” to 5 “totally agree” was used.

### Data Analysis

The methodologies used were exploratory factor analysis (EFA) and confirmatory factor analysis (CFA) to validate the measurement scales. The statistical program SPSS 19.0 (Statistical Package for the Social Sciences) was used for the exploratory analysis, and AMOS 20.0 (Analysis of Moment Structures) software was used for the confirmatory analysis.

#### Validation of the Measurement Model

The psychometric properties of the scales (Anderson and Gerbing, 1988), reliability and unidimensionality, were analyzed first by means of an exploratory analysis as a prior phase to the application of a CFA.

First, the reliability of the scales or the degree to which a measurement is free of random errors was analyzed. It is measured through two indicators (Kunder–Richardson method): (1) item–total correlation >0.3 (Nurosis, 1993) and Cronbach's alpha >0.7. The latter evaluates the internal consistency of the scale through the correlation of each of the variables with the rest of the scale. Once contrasted, unidimensionality is analyzed, which allows us to obtain the explained variance percentage, the factor loading of each indicator, and to observe if they load on more than one factor, loadings >0.05 (Hair et al., 1999), and the percentage of the explained variance >50%. First, the EFA of principal components with varimax rotation (Bagozzi and Baumgartner, 1994) is applied, and based on these results, the CFA allowed us to examine the measurement model (reliability and validity of measures), the structural model, and the global model of each of the scales. To examine the fit of the structural measurement model, it is confirmed that the critical ratio for regression weight must exceed ±1.96 and the standard regression weight (β) >0.5 (Jöreskog and Sörbom, 1993). To examine the global model, the goodness-of-fit indices (GFIs) of the model are observed (Lévy-Mangin and Varela-Mallou, 2006): (1) absolute fit indices [GFI > 09, root mean square error of approximation (RMSEA) <0.08], incremental adjustment indices [normed fit index (NFI), comparative fit index (CFI), adjusted goodness-of-fit index (AGFI), they must all be >0.9], parsimony indices [parsimonious goodness-of-fit index (PGFI)], the higher the value, the greater the parsimony of the model. Values between 2 and 3 are recommended (Jöreskog and Sörbom, 1993) for normalized χ^2^ (χ^2^/df). To finish, the reliability is estimated again through composite reliability (CR) >0.7: The higher the reliability, the greater the internal consistency of its indicators and variance extracted (AVE) >0.5; this measures the total amount of variance of the indicators that is taken into account by the latent construct.

#### Estimation of Structural Equation Modeling (SEM)

The SEM technique or covariance structure model was used to test the proposed structural model. The statistical program AMOS 20.0 allowed us to test the causal relationships proposed in the theoretical model (β standard regression weight and critical coefficient > ±1.96). Taking into account the sample size and the infringement of the assumption of normal distribution of the observable variables (Kolmogorov–Smirnov test and analysis of multivariate kurtosis and critical ratio), the method of maximum likelihood (ML) (bootstrap 500 samples) was chosen for the estimation of the model. Lévy-Mangin and Varela-Mallou (2006, p. 163–166) propose four stages “parameter estimation, adjustment evaluation, re-specification of the model, and interpretation of results.” The structural and global model was evaluated through the GFIs already discussed. The coefficient of determination for each structural equation is represented by *R*^2^, which indicates the proportion of variance explained by the exogenous factor in each of the endogenous factors (Lévy-Mangin and Varela-Mallou, 2006, p. 245).

## Results

### Measurement Model

First, the reliability analysis of the measurement scales (item–total correlation and Cronbach's α estimation) is performed in order to examine the internal consistency of each of the measurement instruments and to determine if it is necessary to eliminate any item. At the same time, it is evaluated whether the items that measure each construct do so in a consistent and stable manner, as well as whether they are free from systematic and random errors. An adequate internal consistency of them is corroborated: total-item correlation >0.3, except IC6, being necessary to eliminate this item; Cronbach's α is higher than the recommended minimum of 0.7 (Table 1).

**Table 1:** Descriptive findings and exploratory factor analysis (reliability and validity of scales).

**Constructs included SEM**		**Scale items^a^**	**Mean**	**(s.d.)^b^**	**Item-total**	**Exploratory factor analysis**	
						**Loadings**	**Bartlett's test of sphericity Kaiser–Meyer–Oklin index^c^**
Perceived quality	Facilities (α Cronbach: 0.765)	F1 F2 F3 F4 F5	2.47 1.88 2.84 2.04 3.48	1.06 0.81 0.90 0.99 1.10	0.575 0.652 0.498 0.606 0.545	0.763 0.817 0.663 0.788 0.624	χ^2^(sig.): 312.649 (0.000) KMO: 0.784 Measure of simple adequacy: (0.795–0.821) % Variance: 53.980
	Service staff (α Cronbach: 0.931)	SS1 SS2 SS3 SS4 SS5 SS6 SS7 SS8 SS9 SS10 SS11	3.95 3.46 3.25 3.04 2.90 3.09 2.95 3.03 2.98 2,76 2.95	0.97 1.18 1,07 1,16 1.15 1.18 1.17 1.11 1.09 1.05 1.12	0.474 0.661 0.760 0.714 0.790 0.850 0.832 0.688 0.518 0.769 0.790	0.535 0.721 0.807 0.773 0.837 0.886 0.873 0.750 0.585 0.819 0.842	χ^2^(sig.): 1694.210 (0.000) KMO: 0.936 Measure of simple adequacy: (0.932–0.912) % Variance: 59.889
	Teacher's attitudes and behavior (α Cronbach: 0.944)	TAB1 TAB2 TAB3 TAB4 TAB5 TAB6 TAB7 TAB8 TAB9 TAB10 TAB11 TAB12	3.23 3.00 3.23 3.25 3.29 4.06 3.26 3.32 3.25 3.27 3.00 3.12	1.05 1.07 1.01 0.99 1.03 0.93 1.01 0.99 1.02 1.02 1.04 1.02	0.712 0.675 0.696 0.792 0.791 0.496 0.724 0.765 0.847 0.815 0.802 0.785	0.756 0.727 0.743 0.831 0.833 0.552 0.772 0.811 0.879 0.855 0.845 0.829	χ^2^(sig.): 2114.096 (0.000) KMO: 0.935 Measure of simple adequacy: (0.902–0.932) % Variance: 62.502
	Competence of teachers (α Cronbach: 0.933)	CT1 CT2 CT3 CT4 CT5 CT6 CT7 CT8	3.43 3.18 3.21 3.08 3.03 3.04 3.08 3.24	1.00 1.02 1.05 0.95 0.96 1.04 0.99 1.02	0.773 0.758 0.795 0.811 0.797 0.796 0.780 0.631	0.829 0.815 0.848 0.865 0.854 0.851 0.840 0.704	χ^2^(sig.): 1396.424 (0.000) KMO: 0.906 Measure of simple adequacy: (0.904–0.947) % Variance: 68.434
	Career opportunity (α Cronbach: 0.855)	CO1 CO2 CO3	2.33 2.38 2.15	0.92 1.06 1.04	0.701 0.739 0.751	0.865 0.886 0.893	χ^2^(sig.): 299.999 (0.000) KMO: 0.730 Measure of simple adequacy: (0.768–0.708) % Variance: 77.695
	Perceived value (α Cronbach: 0.799)	PV1 PV2 PV3 PV4	2.78 3.43 3.22 3.70	0.99 1.08 1.07 0.97	0.507 0.652 0.696 0.599	0.700 0.824 0.849 0.782	χ^2^(sig.): 283.938 (0.000) KMO: 0.759 Measure of simple adequacy: (0.780–0.774) % Variance: 62.536
	Expectation (α Cronbach: 0.807)	E1 E2 E3	2.90 2.59 2.76	0.95 1.05 0.97	0.658 0.638 0.675	0.852 0.838 0.862	χ^2^(sig.): 219.270 (0.000) KMO: 0.713 Measure of simple adequacy: (0.711–0.695) % Variance: 72.372
	Satisfaction (α Cronbach: 0.914)	S1 S2 S3	3.19 3.00 3.15	1.14 1.06 1.15	0.846 0.786 0.854	0.933 0.902 0.937	χ^2^(sig.): 478.371 (0.000) KMO: 0.747 Measure of simple adequacy: (0.721–0.710) % Variance: 85.405
	Loyalty (α Cronbach: 0.914)	L1 L2 L3 L4	2.44 2.80 3.09 2.85	1.17 1.14 1.12 1.14	0.753 0.850 0.754 0.857	0.859 0.921 0.860 0.925	χ^2^(sig.): 640.756 (0.000) KMO: 0.840 Measure of simple adequacy: (0.879–0.806) % Variance: 79.537

a *The items listed in this table have been summarized for ease of presentation and comprehension*.

b *s.d., standard deviation*.

c *Tests that show that the data obtained through the questionnaire are adequate to perform the factor analysis (requirements: Bartlett's sphericity test χ^2^ (sig. <0.5), KMO > 0.7 median, >0.8 and >0.9 very good, MSA = unacceptable for values below 0.5)*.

Source: Authors' own data.

Second, unidimensionality is confirmed by applying EFA, in order to determine the percentage of explained variance and the factor loading of each indicator. The method used is principal axis analysis with varimax rotation (Bagozzi and Baumgartner, 1994), and those indicators with factor loadings <0.5 (Hair et al., 1999) are eliminated. All measurement scales are checked to be unidimensional.

As a last step, CFA is applied to confirm the unidimensionality results. In the specific case of the perceived quality scale, the EFA finds that the items that measure each construct (they are considered taking into account the previous literature) do so appropriately. In addition, taking into account that the perceived quality was treated as a multidimensional construct or as independent measurement constructs of this concept in the previous literature, in this research, by following Hair et al. (1999), the strategy of rival models (Table 2) is developed, in order to check for multidimensionality. Model 1 is proposed, in which all items load on a single variable (perceived quality), which is compared to two first-order Model 2 with five quality dimensions (oblique and orthogonal). Model 2 (oblique) of the first order is shown to have a better fit than Models 1 and 2 (orthogonal), which is re-specified to improve the fit (Model 3), and is compared to a second-order model (Model 4). The results confirm that the optimal measurement model is Model 4 of the second-order (items CI3, CI5, CA3, CA4, and CPS1 are eliminated, as they do not have significant β). The model has good measures of absolute, incremental, and parsimony adjustment.

**Table 2:** Fit indices for quality perceived scale.

**Models**	**χ^2^**	**df**	**χ^2^ (df)**	**GFI**	**AGFI**	**PGFI**	**TLI**	**CFI**	**RMSEA**
Model 1 (1 variable: 39 items)	3487.352	665	5.244	0.433	0.369	0.389	0.543	0.568	0.138
Model 2: first-order (5 variables: 39 items) (orthogonal)	2306.476	702	3.286	0.633	0.592	0.570	0.752	0.765	0.101
Model 2: first-order (5 variables: 39 items) (oblique)	1856.567	692	2.683	0.684	0.643	0.607	0.817	0.829	0.87
Model 3 (model 2 re-specified: 5 variables and 34 items)	1109.311	536	2.070	0.783	0.745	0.666	0.899	0.909	0.069
Model 4: second-order (model 2 re-specified: 6 variables and 34 items)	1038.074	508	2.043	0.788	0.752	0.673	0.904	0.913	0.068
Recommended minimums	Low values	Low values	Recommended values between 2 and 3	>0.9	>0.9	Higher values preferable	>0.9	Recommended values close to 1	Values <0.08

*Source: Authors' own data*.

Table 3 shows the results of the CFA of the scales of the optimal measurement model (perceived quality and joint scale of satisfaction, perceived value, expectations, and loyalty). All items have β > 0.50 and are significant (critical coefficient > ±1.96). The two models presented show good measures of absolute, incremental, and parsimony adjustment.

**Table 3:** Reliability and confirmatory factor analysis.

**Scales**		**β**	**CR**	**AV**	**Confirmatory factory analysis/composite reliability test**	**Scales**	**β**	**CR**	**AV**	**Confirmatory factory analysis/composite reliability test**
Perceived Quality	Facilities		0.81	0.58	$\chi_{\left(\text{df}5\right)}^{2}$ = 1038.074 (*p* = 0.000), GFI = 0.788, AGFI = 0.752, CFI = 0.913, RMSEA = 0.068, χ^2^ normalized (χ^2^/df) = 2.043	Satisfaction		0.93	0.81	$\chi_{\left(\text{df}5\right)}^{2}$ = 161.727 (*p* = 0.000), GFI = 0.910, AGFI = 0.863, CFI = 0.960, RMSEA = 0.078, χ^2^-normalized (χ^2^/df) = 2.344
	F1	0.701								
						S1	0.250			
						S2	0.344			
	F2	0.816				S3	0.205			
	F4	0.746								
	Service staff		0.90	0.53		Perceived value		0.82	0.54	
	SS2	0.658				PV1	0.532			
	SS3	0.737				PV2	0.611			
	SS4	0.737								
						PV3	0.407			
	SS5	0.801				PV4	0.627			
	SS6	0.850								
	SS7	0.877								
	SS8	0.750								
	SS9	0.549								
	SS10	0.800								
	SS11	0.852								
	Teacher's attitudes and behavior		0.93	0.62		Expectation		0.85	0.65	
	TAB1	0.712				E1	0.334			
	TAB2	0.682				E2	0.529			
	TAB4	0.798				E3	0.389			
	TAB5	0.812								
	TAB7	0.754								
	TAB8	0.807								
	TAB9	0.878								
	TAB10	0.852								
	TAB11	0.839								
	TAB12	0.814								
	Competence of teachers		0.92	0.61		Loyalty		0.93	0.76	
	CT1	0.730				L1	0.516			
	CT2	0.725				L2	0.235			
						L3	0.434			
						L4	0.233			
	CT3	0.787								
	CT4	0.839								
	CT5	0.839								
	CT6	0.844								
	CT7	0.830								
	CT8	0.650								
	Career opportunity		0.86	0.66						
	CO1	0.773								
	CO2	0.855								
	CO3	0.818								

*β, standard regression weight; CR, composite reliability; AV, average variance*.

*p < 0.001*.

*Source: Authors' own data*.

At this point, it is necessary to check the reliability of the measurement scales again. It is measured through average variance (AV) and CR, which must be >0.5 and >0.7, respectively (Table 4) (Bagozzi and Yi, 1988; Hair et al., 1999), and it is corroborated. The content validity was ensured by the literature review carried out, as well as by the pretest carried out, and convergent validity is corroborated as β > 0.5 and statistically significant (Student's *t*-test > ±1.96) and AVE > 0.5 (Table 4).

**Table 4:** Correlation matrix and discriminant validity.

	**Square root AV**	**(1)**	**(2)**	**(3)**	**(4)**	**(5)**	**(6)**	**(7)**	**(8)**	**(9)**
Facilities (1)	0.76	**0.765**^a^	0.355^b^	0.415	0.452	0.492	0.456	0.364	0.275	0.419
Service staff (2)	0.72		**0.931**	0.467	0.469	0.310	0.509	0.417	0.373	0.441
Teacher's attitudes and behavior (3)	0.78			**0.944**	0.778	0.4463	0.654	0.621	0.615	0.617
Competence of teachers (4)	0.78				**0.933**	0.522	0.656	0.524	0.537	0.522
Career opportunity (5)	0.81					**0.855**	0.521	0.479	0.407	0.465
Perceived value (6)	0.73						**0.807**	0.627 0.168^c^ (0.289–0.533)	0.712 0.337 (0.421–0.741)	0.643 0.227 (0.333–0.621)
Expectation (7)	0.80							**0.914**	0.734 0.418 (0.483–0.811)	0.670 0.305 (0.401–0.705)
Satisfaction (8)	0.90								**0.799**	0.800 0.695 (0.632–0.936)
Loyalty (9)	0.87									**0.914**

a *Shown in boldface on the main diagonal are the Cronbach's alpha for each scale, which should be higher than the correlation between that scale and the rest*.

b *Interscale correlation*.

c *The squared correlation between pairs of factors (less than AVE) and confidence interval for the estimated correlations, ± twice the standard error, does not include the value of 1*.

*All significant at p-value < 0.01*.

*Source: Authors' own data*.

To conclude, discriminant validity was analyzed by examining three indicators: (1) confirmed if Cronbach's alpha of each scale is higher than any of the correlations between that scale and the rest, which was proven, and (2) whether interfactor correlations are less than the square root of the AV extracted (Fornell and Larcker, 1981; Chin, 1998), and (3) none of the confidence intervals contains the unit (Bagozzi and Yi, 1988). Taking into account the results, discriminant validity is confirmed.

### Analysis of the Structural Models

**Figure 2:**
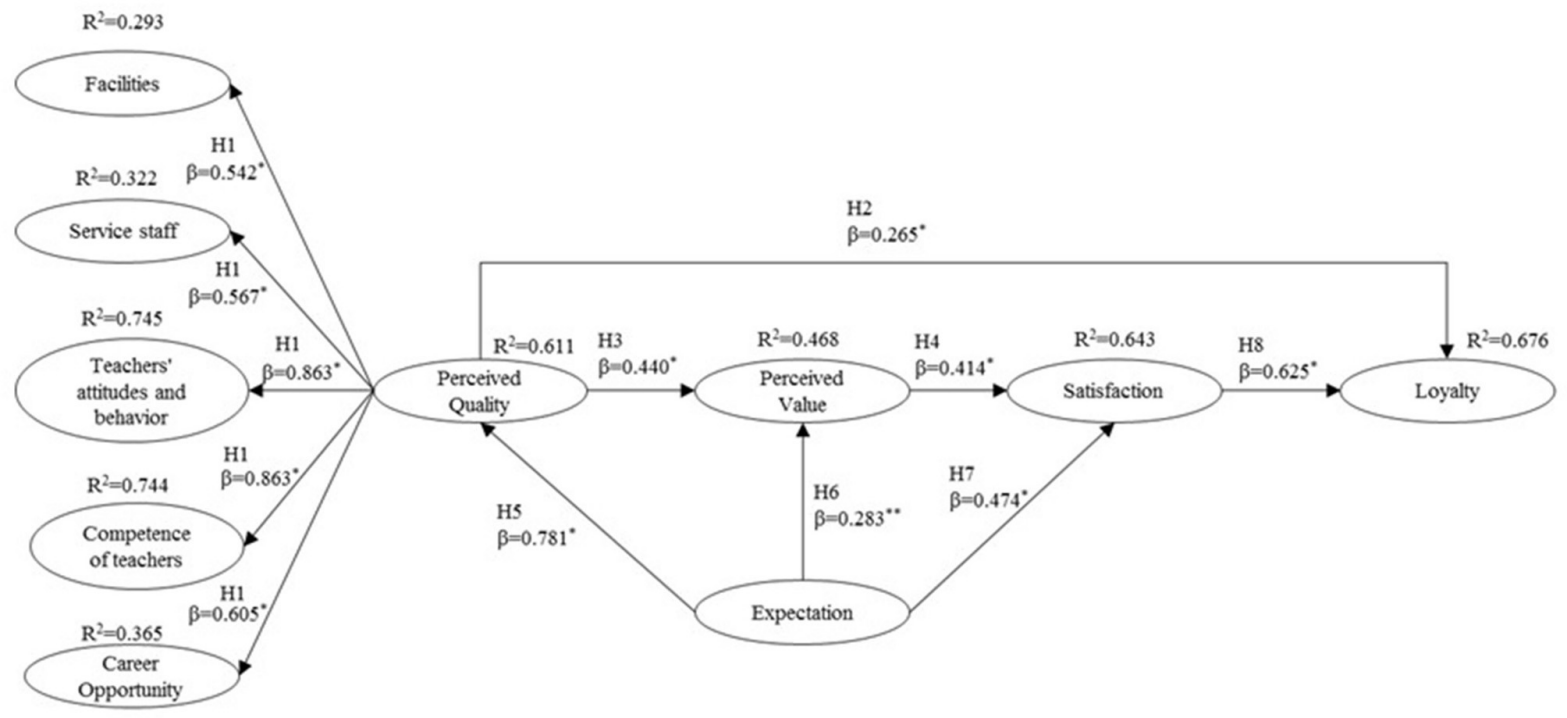
Structural model. *p < 0.001;**p < 0.01. $\chi_{\left(\text{df}7\right)}^{2}$ = 65.807 (*p* = 0.000), GFI = 0.940, AGFI = 0.887, CFI = 0.966, RMSEA = 0.088, χ^2^-normalized (χ^2^/df) = 2.742.

The research hypotheses in the proposed theoretical model were tested (Figure 2). The standardized coefficients (β) that show the weights of the direct effects of one variable on another and the direction (hypothesis) are all significant at the *p* < 0.001, 0.01 level, and therefore, all the hypotheses proposed are corroborated. The structural model has good adjustment measures, and all the indices have values within the recommended limits. The model explains 67.6% (*R*^2^) of loyalty.

**Table:** Direct, indirect, and total effects.

	**Perceived quality**		**Perceived value**		**Expectation**		**Satisfaction**	
**Construct**	**Direct**	**Indirect**	**Direct**	**Indirect**	**Direct**	**Indirect**	**Direct**	**Indirect**
Loyalty	0.265	0.114	–	0.259	–	0.667	0.625	–
Total effects	0.380		0.259		0.667		0.625	
Perceived value	0.440	–	–	0.283	0.344	–	–
Total effects	0.440		–		0.627		–	
Perceived quality	–	–	–	–	0.781	–	–	–
Total effects	–		–		0.781		–	
Satisfaction	–	0.183	0.414	–	0.474	0.260	–	–
Total effects	0.183		0.414		0.734		–	

*Source: Authors' own data*.

## Discussion

The results of the structural model (Figure 2), which studies the direct influence of perceived quality on loyalty and indirectly through perceived value and satisfaction, support the explanatory capacity of the proposed theoretical model. The student's loyalty dimension with *R*^2^ = 0.676 > 0.67 (criteria proposed by Hu and Bentler, 1997) allows us to confirm that its explanatory capacity is strong, and the model explains 67.7% of loyalty. Regarding the proposed hypotheses, they are corroborated with significant β (*p* > 0.001 and 0.01). β indicates the relative importance of the dependent variable.

Hypothesis H1, which proposes that perceived service quality is a reflective and multidimensional construct with five dimensions, is corroborated. The dimensions of teachers' attitudes and behavior and competence of teachers show a high explanatory capacity in the model (*R*^2^ = 0.745 and 0.744, respectively). The explanatory capacity of the facilities (*R*^2^ = 0.293), service staff (*R*^2^ = 0.322), and career opportunity (*R*^2^ = 0.365) dimensions is weaker. These results are partially corroborated by the study of De la Fuente Mella et al. (2010), from which the scale was obtained. These authors consider seven dimensions, including reputation and other services, which in this research are included in the five dimensions considered. The main difference is that these authors considered the constructs separately as indicators of perceived service quality, and in this study, they are presented and validated as reflective indicators of the perceived quality construct. Taking into account that currently there is no consensus on the dimensions (Sultan and Wong, 2012) or on the best way to define and measure service quality (Clewes, 2003), the comparison of the results of this research regarding this construct is complex. However, there are many investigations that consider that the perceived quality construct is multidimensional and reflective. Among them, Subrahmanyam and Shekhar (2014) and Annamdevula and Bellamkonda (2016), that considered six dimensions (teaching, administrative services, academic facilities, campus infrastructure, support services, and internationalization) and Huili and Jing (2012), that took into account four (material resources, teacher resources, campus environment and quality of the results).

Regarding causal relationships, perceived service quality influences directly (β = 0.265, *p* < 0.001) (Hypothesis H2) and indirectly in building student's loyalty. The mediating variables between perceived quality and loyalty are perceived value (Hypothesis H3) with β = 0.440, *p* < 0.001, and satisfaction, as it is influenced by perceived value (Hypothesis H4) (β = 0.414, *p* < 0.001). Finally, satisfaction influences loyalty directly (H8) (β = 0.625, *p* < 0.001). The causal relationship proposed by Hypothesis H2 (perceived quality → loyalty) is also corroborated by the research carried out by Chandra et al. (2019) and Hassan et al. (2019). The relationship between perceived quality → perceived value (H3) is corroborated by LeBlanc and Nguyen (1997); Alves and Raposo (2007); Clemes et al. (2008, 2013); Huili and Jing (2012); Kwok et al. (2017), and de Oliveira Silva et al. (2020). The relationship between perceived value → satisfaction (H4) is corroborated by LeBlanc and Nguyen (1997), Alves and Raposo (2007), Clemes et al. (2008), Huili and Jing (2012), Clemes et al. (2013), Kwok et al. (2017), and de Oliveira Silva et al. (2020). Finally, the relationship between satisfaction → loyalty (H8) is also corroborated among others by Chen et al. (2005), Alves and Raposo (2007), Tsuji et al. (2007), Clemes et al. (2008), Huili and Jing (2012), Clemes et al. (2013), Sultana and Momen (2017), and de Oliveira Silva et al. (2020).

Regarding students' expectations, these influence perceived quality (H5), perceived value (H6), and satisfaction (H7). The relationship between expectations and perceived quality (β = 0.781, *p* < 0.001) is strong, as well as with satisfaction (β = 0.474, *p* < 0.001), being weaker with perceived value (β = 0.283, *p* < 0.01). These results are corroborated by the investigations of Alves and Raposo (2007) and Huili and Jing (2012).

Considering the direct and indirect effects, the key variables to increase loyalty are expectations and satisfaction with a total effect of 0.667 and 0.625, respectively. Regarding expectations, its influence is indirect through perceived quality (0.781), perceived value (0.627), and satisfaction (0.734). Perceived value is the construct that least affects loyalty with a total effect of 0.259, but its effect is very important in the formation of satisfaction (with a total effect of 0.414). Perceived quality affects loyalty moderately (0.380). Regarding satisfaction, it is formed from the effect of the indirect effect of quality (0.183), the direct effect of perceived value (0.414), and the direct effect (0.474) and indirect effect (0.260) with a total effect of 0.734 of expectations.

## Conclusions and Implications

The structural model proposed and empirically validated in this research confirms that the key variables to improve student's loyalty and influence their behavior regarding continuing to study (Master's) in the center or recommending it to other people are expectations and the satisfaction perceived by students; both variables constitute an important source of competitive advantage. It is also observed that expectations are one of the three key variables to achieve satisfaction, together with service quality and perceived value. This research and its results allow us to understand the relationship between expectations and satisfaction with loyalty and to identify the antecedent variables of satisfaction (perceived service quality, perceived value, and expectations), as well as to obtain evidence of the importance of expectations within the model, for the formation of both perceived quality and satisfaction.

These results are highly useful for higher education center managers, always taking into account the characteristics of each center since they allow them to observe which variables are the most important to achieve their objective of retaining current students and that these students serve as positive communication channels for attracting new students. In this regard, they must focus their efforts and implement the necessary strategies to adapt expectations to the service quality offered by the center, especially regarding the attitude and behavior of their teachers, as well as improving their skills, and it is also very important to convey information to their students about their career opportunities, once they have graduated. They should also focus on improving the satisfaction perceived by the students, without forgetting that the expectations that the center has conveyed to its current or potential students are very important since they affect both the perceived quality of the students and their satisfaction in a highly significant way and in return for the loyalty of their students.

The limitations of the study are as follows: The research is carried out at a specific point in time (cross-sectional), and the population and the sample refer to a single higher education center located in a specific country, namely, Spain. This limits the generalizability of the results to a certain extent. The third limitation is related to the use of a structured questionnaire that limits the responses of the respondents to the questions asked, a limitation that has been overcome by considering the results of the CMB test. These limitations can be overcome by expanding the study to a greater number of university centers, as well as opening the geographical area to other countries, allowing the behavior of the model to be compared in environments with different structural characteristics.

## Data Availability Statement

The raw data supporting the conclusions of this article will be made available by the authors, without undue reservation.

## Ethics Statement

Ethical review and approval was not required for the study on human participants in accordance with the local legislation and institutional requirements. Written informed consent from the participants was not required to participate in this study in accordance with the national legislation and the institutional requirements.

## Author Contributions

MdR-R, JÁ-G, NKM, and AD-S: conceptualization, investigation, methodology, formal analysis, writing-original draft, preparation, and writing-review & editing. All authors have read and agreed to the published version of the manuscript.

## Conflict of Interest

The authors declare that the research was conducted in the absence of any commercial or financial relationships that could be construed as a potential conflict of interest.
