# Supplementary material for: Influence of the Quality Perceived of Service of a Higher Education Center on the Loyalty of Students
Source: Front Psychol. 2021 Jun 7;12:671407. doi: 10.3389/fpsyg.2021.671407 (PMC8216109; doi:10.3389/fpsyg.2021.671407)
Supplement: Supplementary file 1 [file Data_Sheet_1.docx]

**APPENDIX. Measurement scales**

| Perceived Quality |
| --- |
| Facilities  F1- The conditions of the classrooms of this Faculty are adequate for teaching  F2- The equipment (laboratories, computer rooms, etc.) of this Faculty is modern  F3- The bibliographic funds available of this faculty are sufficient  F4- The physical facilities of this Faculty are comfortable and cozy  F5- The physical facilities of this Faculty are clean and tidy  F6- The laboratories and their equipment are suitable for the facilities to be developed  Service staff  SS1- The service staff of this Faculty has a neat appearance  SS2- The service staff of this Faculty is always friendly  SS3- The service staff of this Faculty gives you personalized attention  SS4- The service staff of this Faculty informs you precisely when each service will end  SS5- The service staff of this Faculty serves you quickly and agilely  SS6- The behaviour of the service staff of this Faculty conveys confidence and security  SS7- When you have a problem, the service staff of this Faculty shows a sincere interest in solving it  SS8- The service staff of this Faculty has sufficient knowledge to answer any queries you have  SS9- The service staff of this Faculty is never too busy to answer your questions  SS10- The service staff of this Faculty understands your particular needs  SS11- The service staff of this Faculty is always ready to help you, making an effort to meet your needs  Teachers' attitudes and behaviour  TAB1- The teachers of this Faculty are concerned with student learning  TAB2- The teachers of this Faculty motivate their students  TAB3- The teachers of this Faculty encourage student participation  TAB4- The teachers of this Faculty are available to guide the student when necessary  TAB5- There is a fluid and spontaneous communication between teachers and students  TAB6- The teachers of this Faculty have a neat appearance  TAB7- The teachers of this Faculty are always kind to you  TAB8- The teachers of this Faculty give personalized attention  TAB9- The behaviour of the teachers of this Faculty conveys confidence and security  TAB10- When you have a problem, the teachers of this Faculty show a sincere interest in solving it  TAB11- The teachers of this Faculty understand your particular needs  TAB12- The teachers of this Faculty are always ready to help you to satisfy your needs  Competence of teachers  CT1- The teaching staff of this Faculty has a sufficient theoretical knowledge level  CT2- The teaching staff of this Faculty has a sufficient practical knowledge level  CT3- The teaching staff of this Faculty is up to date on their knowledge  CT4- The teaching staff of this Faculty is able to transmit their knowledge adequately  CT5- The teaching staff of this Faculty explains the concepts clearly enough  CT6- The teaching staff of this Faculty uses useful material for students  CT7- The teaching staff of this Faculty uses the appropriate teaching methods  CT8- The teaching staff of this Faculty complies with the work plan  Career Opportunity  CO1- It is easy for graduates to find work  CO2- Excellent information on career opportunities  CO3- The Faculty helps graduates to find their first job |
| Perceived Value |
| PV1- Taking into account the time, effort and money spent, you consider that the value received when completing your studies is adequate  PV2- You consider that the studies carried out have been a good choice  VV3- Comparing everything you have obtained during the studies completed (training, service, environment, relationships, etc.) with all the sacrifices made (time and effort spent, money, etc.), your assessment of these studies is positive  PV4- Taking these studies has value for your professional career |
| Expectations |
| E1- The Faculty meets my needs  E2- The idea that I had formed about the Faculty corresponds to the reality lived  E3- In general terms, the faculty offers me high levels of confidence |
| Satisfaction |
| S1- The Faculty meets my needs  S2- The idea that I had formed about the Faculty corresponds to the reality lived  S3- In general terms, the faculty offers me high levels of confidence |
| Loyalty |
| L1- If you had to do other courses, conferences or professional improvement studies, you would surely consider the Faculty as the first option  L2- If someone asked you for advice, you would recommend the Faculty  L3- If you had the opportunity, you would discuss positive things about the Faculty with your friends and family  L4- You would encourage family and friends to study at this Faculty |
